# GBS-DP: a bioinformatics pipeline for processing data coming from genotyping by sequencing

**DOI:** 10.18699/VJGB-23-86

**Published:** 2023-12

**Authors:** A.Y. Pronozin, E.A. Salina, D.A. Afonnikov

**Affiliations:** Institute of Cytology and Genetics of the Siberian Branch of the Russian Academy of Sciences, Novosibirsk, Russia Kurchatov Genomic Center of ICG SB RAS, Novosibirsk, Russia; Institute of Cytology and Genetics of the Siberian Branch of the Russian Academy of Sciences, Novosibirsk, Russia Kurchatov Genomic Center of ICG SB RAS, Novosibirsk, Russia Novosibirsk State Agrarian University, Novosibirsk, Russia; Institute of Cytology and Genetics of the Siberian Branch of the Russian Academy of Sciences, Novosibirsk, Russia Kurchatov Genomic Center of ICG SB RAS, Novosibirsk, Russia Novosibirsk State University, Novosibirsk, Russia

**Keywords:** genotyping by sequencing (GBS), bioinformatic pipeline, hordeum, генотипирование путем секвенирования, биоинформатический конвейер, ячмень

## Abstract

The development of next-generation sequencing technologies has provided new opportunities for genotyping various organisms, including plants. Genotyping by sequencing (GBS) is used to identify genetic variability more rapidly, and is more cost-effective than whole-genome sequencing. GBS has demonstrated its reliability and flexibility for a number of plant species and populations. It has been applied to genetic mapping, molecular marker discovery, genomic selection, genetic diversity studies, variety identification, conservation biology and evolutionary studies. However, reduction in sequencing time and cost has led to the need to develop efficient bioinformatics analyses for an ever-expanding amount of sequenced data. Bioinformatics pipelines for GBS data analysis serve the purpose. Due to the similarity of data processing steps, existing pipelines are mainly characterised by a combination of software packages specifically selected either to process data for certain organisms or to process data from any organisms. However, despite the usage of efficient software packages, these pipelines have some disadvantages. For example, there is a lack of process automation (in some pipelines, each step must be started manually), which significantly reduces the performance of the analysis. In the majority of pipelines, there is no possibility of automatic installation of all necessary software packages; for most of them, it is also impossible to switch off unnecessary or completed steps. In the present work, we have developed a GBS-DP bioinformatics pipeline for GBS data analysis. The pipeline can be applied for various species. The pipeline is implemented using the Snakemake workflow engine. This implementation allows fully automating the process of calculation and installation of the necessary software packages. Our pipeline is able to perform analysis of large datasets (more than 400 samples).

## Abstract (ru)

Развитие технологий секвенирования нового поколения открыло новые возможности для генотипирования различных организмов, включая растения. Метод генотипирования путем секвенирования (GBS) применяется для идентификации генетической изменчивости и более быстрого генотипирования образцов, а также является более экономически эффективным методом в сравнении с полногеномным секвенированием. GBS продемонстрировал свою надежность и гибкость для ряда видов и популяций растений. Этот метод был применен для генетического картирования, выявления молекулярных маркеров, геномной селекции, в исследовании генетического разнообразия, идентификации сортов, а также в исследованиях в области биологии охраны природы и эволюционной экологии. Однако сокращение времени и стоимости секвенирования привело к необходимости разработки качественного биоинформатического анализа для постоянно расширяющегося количества секвенированных данных. Для этих целей были разработаны биоинформатические конвейеры анализа данных, полученных методом GBS. Вследствие схожести этапов обработки существующие конвейеры в основном различаются комбинацией программных пакетов, специфически подобранных для обработки данных как для определенных, так и для любых организмов. Несмотря на качественно подобранные пакеты программ, конвейеры имеют некоторые недостатки, например отсутствие возможности автоматизации процесса расчета (каждый этап нужно запускать вручную), что значительно снижает скорость исследования. В большинстве конвейеров отсутствует возможность автоматической установки всех необходимых программных пакетов, а также нет возможности отключения ненужного или пройденного этапа. В настоящей работе нами был разработан биоинформатический конвейер GBS-DP для анализа данных, полученных методом GBS. Конвейер применим для любых видов организмов. Реализация конвейера на платформе Snakemake позволила полностью автоматизировать процесс расчета и установки необходимых программных пакетов. Конвейер позволяет обрабатывать большие объемы данных (более 400 образцов).

## Introduction

Genetic diversity is the most important basis for studying plant resistance to biotic and abiotic stresses and for developing new highly adaptive and high-yielding crop sorts. Study of genetic diversity is performed using various methods of DNA analysis. To date, one of the most advanced methods is the use of molecular markers (Kanukova et al., 2019). Molecular markers (DNA markers) are genetic markers analysed at the nucleotide level (Khlestkina, 2013). Their use allows to identify genetic diversity of populations, subspecies, species, allowing to effectively determine loci controlling economically valuable plant traits even at the initial stage of breeding (Sukhareva, Kuluev, 2018)

Some of the most convenient DNA markers for genetic analysis are SNP markers (Khlestkina, 2013). SNP (Single- Nucleotide Polymorphism) is a single-nucleotide position in genomic DNA for which different sequence variations (alleles) occur in the population (Sukhareva, Kuluev, 2018). SNPs are widely used for allelic polymorphism studies, seed purity testing, haplotype and pedigree analyses, as well as for genotyping and construction of genetic maps.

Obtaining SNP marker information is now possible for any plant at a whole genome scale through the use of next-generation high-throughput sequencing technologies. Identification of SNPs is possible using whole-genome sequencing (WGS) and genotyping by sequencing (GBS) strategies (Scheben et al., 2017). The aim of whole-genome sequencing is to obtain short random fragments (reads) of the whole genome DNA. This allows estimating DNA variation by aligning fragments to a reference genome or by genomic DNA de novo assembly. This can be challenging and expensive; price per genome exceeds $2000, also depending on the size and complexity of the genome, the desired level of completeness and computational resources (Narum et al., 2013). For example, sequencing a complete barley genome to the chromosome level costs around $60,000 (Monat et al., 2019). There are also specific methods of whole-genome sequencing with lower read depths that cost much less, $100–$400 per genome. However, according to the authors (Bimber et al., 2016), the accuracy of the resulting genotype data is decreased.

The genotyping by sequencing method is faster and more cost-efficient than the whole-genome sequencing method. For example, the cost of single barley genome sequencing by fragments in a GBS experiment does not exceed $30 (Monat et al., 2019). Two sequencing strategies can be applied in the GBS experiments. The first one uses site-specific restriction enzymes for fragmentation of DNA samples, after which sequencing of the resulting fragments is performed (Glaubitz et al., 2014). In the second method, unique adapter sequences are ligated to both ends of DNA fragments during library preparation (Elshire et al., 2011). Due to the fact that DNA fragments are only sequenced in the region of restriction sites, the GBS method does not sequence the full genome DNA sequence. This makes the sequencing process much cheaper. However, the number of SNPs that can be identified is lower than that obtained with whole-genome sequencing. Nevertheless, the amount of data obtained using the GBS method is sufficient to characterise the genetic diversity of agricultural plant populations with acceptable accuracy.

The GBS method has demonstrated its reliability and flexibility for a number of plant species and populations. GBS has been applied to the identification of molecular markers for genetic mapping (Poland et al., 2012), genomic selection (Poland et al., 2012), in genetic diversity studies (Lu et al., 2013; Peterson et al., 2014), variety identification (Wang et al., 2020; Rajendran et al., 2022), and studies in conservation biology and evolutionary ecology (Narum et al., 2013).

The GBS method significantly reduces the cost as well as the time required for sequencing the samples under study. This has led to the development of high-quality bioinformatics methods for the ever-expanding amount of sequenced data. To date, a number of bioinformatics pipelines for analyzing the data generated by GBS experiments have been developed. The workflows for existing pipelines of the GBS data analysis are similar and include raw reads preprocessing, data demultiplexing (if needed), mapping reads to a reference genome, SNP identification and analysis of genetic diversity.

The reads mapping step depends on the presence or absence of the reference genome sequence. In the first case, preprocessed reads are aligned to a reference genome using existing tools such as bowtie2 or bwa (Glaubitz et al., 2014; Torkamaneh et al., 2017; Wickland et al., 2017). In the absence of reference genome sequences, an additional step of “Mock Reference” sequence generation is applied (Melo et al., 2016). This method clusters reads by their similarity to identify consensus sequences (centroids) on the basis of which the fragments of the genome are assembled (Melo et al., 2016). These fragments of the genomic sequence are used as the reference in subsequent analysis. Due to the similarity of the data processing steps, existing pipelines mainly differ in the software tools combined to perform the analysis. The combination should take into account various genomic characteristics, such as the number of polymorphisms detected, genome complexity, degree of heterozygosity, and the proportion of repetitive sequences in the whole genome. More advanced pipelines allow the selection of parameters for the organisms under study (Torkamaneh et al., 2017; Wickland et al., 2017), whereas earlier pipelines have some limitations. For example, TASSEL needs specification of the sequence length upper limit, which may result in the loss of a significant number of short raw reads (Glaubitz et al., 2014; Melo et al., 2016). Due to the ever-increasing number of sequenced libraries, pipelines must provide the capability to process a large amount of data in a single run. An important aspect of pipelines is the automation of the processing and the simplicity of the software installation

In the present work, we have developed a GBS-DP bioinformatics pipeline for analysing GBS data. This pipeline incorporates the GBS data processing scheme proposed in (Jayakodi et al., 2020). The pipeline is applicable to any organism species. The pipeline can process large amounts of data (more than 400 samples) and is implemented using the Snakemake workflow system (Köster, Rahmann, 2012).

## Materials and methods

**Fig. 1.**
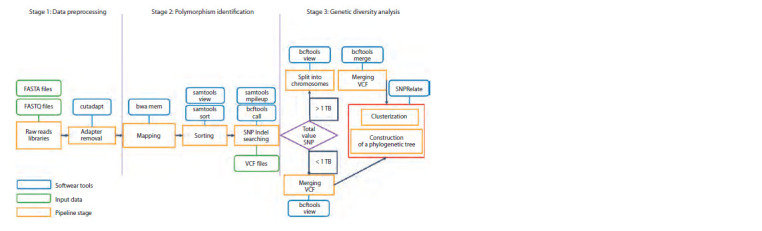
The diagram of the GBS-DP bioinformatics pipeline.

Bioinformatics pipeline for analysing GBS data. The GBS-DP bioinformatics pipeline diagram is shown in Figure 1.

Pipeline input requires the path to the folder with the set of files with raw read sequences and the path to the reference genome file. Files with the read sequences should be in FASTQ format, reference genome, in FASTA format. If the libraries have barcode sequences, they must be demultiplexed beforehand by an external tool (the demultiplexing step is not included in GBS-DP).

The pipeline consists of three main steps: (1) data preprocessing, (2) polymorphism identification, (3) genetic diversity analysis. Data preprocessing includes quality control of raw reads, adapters removal, and construction of reference genome index files. Polymorphism identification includes mapping preprocessed reads to a reference genome, sorting the mapped reads, and searching for single nucleotide polymorphisms. Genetic diversity analysis is performed differently depending on whether the total size of files with polymorphism data exceeds 1 TB. Each stage is described in more detail below.

Data preprocessing. Quality control and adapter removal are performed by cutadapt (Martin, 2011). For the reads of each library, user should provide the list of adapter sequences in the pipeline configuration file.

The reference genome indexing is performed using the bwa tool (‘index’ option) (Li H., 2013).

Polymorphism identification. Mapping of preprocessed reads is performed by the bwa tool (‘mem’ option) (Li H., 2013) with the default parameters “–k 19 –w 100”.

The mapping results are obtained in SAM format, converted into BAM format and sorted using samtools (Danecek et al., 2021) running ‘view’ and ‘sort’ options, respectively. Polymorphisms (SNPs, insertions and deletions (indels)) are identified using the sorted BAM by a combination of samtools (‘mpileup’ option) and bcftools (‘call’ option) (Danecek et al., 2021). It was previously shown using the wheat genome as an example (Yuao et al., 2020) that the samtools/mpileup + bwa-mem software combination used in our pipeline outperforms other combinations of polymorphism mapping and identification software.

Analysis of genetic diversity. The pipeline selects the way of genetic diversity analysis automatically depending on the total size of the VCF files obtained at the previous step.

The corresponding option is selected automatically and associated with increased load on the computer RAM when working with large data (if the total size of the received VCF files exceeds 1 TB). The processing option for data with the total volume less than 1 TB includes three steps. If the total size of files is lower than 1 TB, the pipeline performs the following steps:

1. VCF files containing information about polymorphisms for each sample are indexed using bcftools (‘index’ option) (Danecek et al., 2021).

2. The indexed files are merged into a single VCF file using bcftools (‘merge’ option). This file contains data on polymorphisms of all samples for all chromosomes.

3. The resulting file in VCF format is converted into GDS (Genomic Data Structure) format using the SeqArray package implemented in R (Zheng et al., 2017). This format allows significantly reducing the amount of RAM required for processing the results of polymorphism identification. If the total size of VCF files is greater than 1 TB, the pipeline performs the following steps:

1. Each VCF file with polymorphism data for a specific sample is split by chromosome using bcftools (‘view’ option).

2. The resulting VCF files for each chromosome are indexed using bcftools (‘index’ option).

3. VCF files for each chromosome are merged for all samples. The resulting set of files represent the polymorphism data by chromosome for each sample.

4. VCF files for individual chromosomes are converted to GDS format. The resulting GDS files for each chromosome are then combined into a common GDS file using the snpgdsCombineGeno function of the SNPRelate package (Zheng et al., 2017)

The resulting polymorphism data merged from all samples are used for genetic diversity. It should be noted that important information about SNP distribution in the genomic sequence is represented by the linkage disequilibrium (LD) parameter (Ponomarenko, 2018). Two alleles of different loci are in linkage disequilibrium when the frequency of the haplotype comprising them differs significantly from the frequency expected under random segregation (Gabriel et al., 2002). The value of LD depends on a number of factors: the magnitude and rate of gene drift, genetic admixtures in the population, mutations and recombinations, and population size (Aulchenko, Aksenovich, 2006). LD is usually estimated by the linkage disequilibrium coefficient (D), but this measure is not always convenient because the range of its possible values depends on the frequencies of the alleles to which it refers. This makes it difficult to compare the level of linkage disequilibrium between different pairs of alleles. Thus, the D coefficient is normalised on the basis of the Pearson correlation coefficient r2, which varies from 0 to 1. The closer the value of r2 is to 0, the more likely it is that the identified SNPs are random.

The LD parameter is estimated by the GBS-DP pipeline using the merged file containing polymorphism information for all libraries across all chromosomes in GDS format. The R package SNPRelate (Zheng et al., 2017), function snpgdsLDpruning, is used for LD estimation.

Additionally, principal component analysis is applied for filtered SNPs, which is performed using the R package SNPRelate. The SNPRelate package is also used to build a phylogenetic tree using hierarchical clustering method.

System requirements and installation. The GBS-DP pipeline is implemented using the Snakemake v6.0.0 workflow management system (Köster, Rahmann, 2012), a tool for creating data analysis pipelines implemented in Python. Pipelines created in this environment can be easily scaled for server, cluster, network and cloud environments. Snakemake is compatible with the Conda system, making it easy to install new programs required for the pipeline. The pipeline is designed for the Linux operating system. It requires a minimum of 10 GB of RAM to run (the more data, the more RAM needed). To run the pipeline, user needs to specify parameters in the configuration file. The code and step-by-step instructions for running the pipeline are available at https://github.com/artempronozin95/GBS-DPbioinformatics- pipeline-for-genotyping-by-sequencing-dataprocessing/ tree/main.

Data for test analysis. For the test application of the GBSDP pipeline in the present work, we used project PRJEB39633 from the European Nucleotide Archive (ENA) database (Leinonen et al., 2010), which contains GBS sequencing data for a barley (Hordeum vulgare) population derived from a cross between the six-row barley variety Morex and the mutant line luteostrians-P1 (lst/LST) (Li M. et al., 2021). Libraries were obtained using a combination of MspI and PstI restriction enzymes (Wendler et al., 2015). In total, the PRJEB39633 project contains 679 libraries for 272 genotypes; there is an average of 3 libraries per genotype, so library reads for the same genotype were combined before analysis.

We used the H. vulgare reference genome v. 51 sequence (IBSC_v2) downloaded from the Ensembl plants database (Bolser et al., 2016).

## Results

Supplementary Material1 demonstrates the processing time at different stages of the GBS-DP pipeline execution for different numbers of barley libraries (10, 50, 100, 150, 200 and 272). The characteristics of the computational node are as follows: AMD EPYC 74521 processor, 32 cores, 1 TB memory capacity. For the analysis, we used 100 GB of RAM and 20 processor cores. The longest time was spent on generating a merged file containing polymorphisms. However, it can be seen that the time taken to generate a merged file for 200 libraries is lower than that for 150 libraries; this is due to the usage of big data processing mode, which speeds up the calculation process.


Supplementary Materials are available in the online version of the paper: http://vavilov.elpub.ru/jour/manager/files/Suppl_Pronozin_Engl_27_7.pdf


**Fig. 2.**
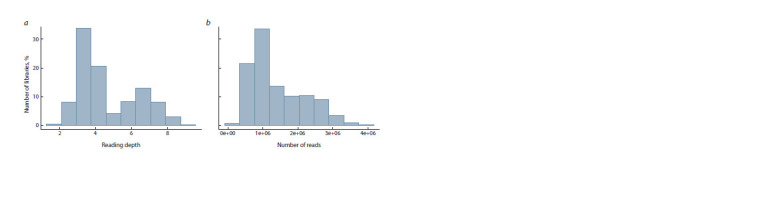
Distribution of average depth of reads mapping (a) and distribution of mapped reads number for libraries (b).

The pipeline provides the results of the basic evaluation of sequenced libraries. The read length for each library is 107 nt. The average read depth (Fig. 2, a) ranges from 2–8,which is an acceptable value for the GBS method. More than 30 % of the libraries contain more than 1,000,000 reads (see Fig. 2, b). On average for one library, the coverage of the barley reference genome (4,225,577,519 nt) with DNA fragments is 3 % of the total length. 

The pipeline also provides the results of the search for polymorphisms between the investigated genotypes. For the 272 samples analysed, 447,409 SNPs were identified. The total number of indels is 46,557. The median value of transitions/transversions = 1.75, indicating the predominance of transitions. The estimate of the LD parameter (r2) is 0.5. After applying the LD filter, 45,402 polymorphic and independent SNPs remained.

**Fig. 3.**
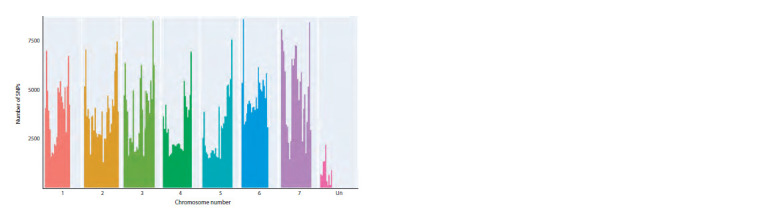
Distribution of detected SNPs in the barley genome. X axis is the coordinates of SNPs on chromosomes, Y axis is the number of SNPs corresponding to these coordinates.

**Fig. 4.**
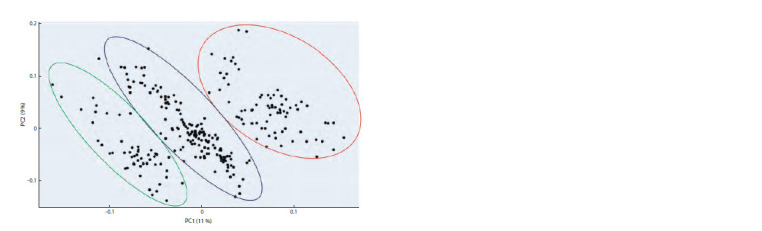
Genotype scatter diagram for the barley population resulting from the cross between the variety Morex and the mutant line luteostrians-P1 (lst/LST) for the two principal components obtained from the genetic diversity analysis by the GBS-DP pipeline The proportion of the total variance is given in parentheses next to the component names.

The distribution of the detected SNPs by chromosome showed that more SNPs were detected for chromosomes 3, 6 and 7 (Fig. 3). The main results of the pipeline are principal component analysis of genotypes based on the detected SNPs (Fig. 4) and construction of a phylogenetic tree. The results of principal component analysis based on 45,402 SNPs allow identifying three distinct clusters within the population. They are clearly distinguished in the scatter plot in the space of the first two components (see Fig. 4). However, the total proportion of variance attributable to these two components is small (20 %), which may indicate an overall high level of genetic diversity in the obtained plant population.

**Fig. 5.**
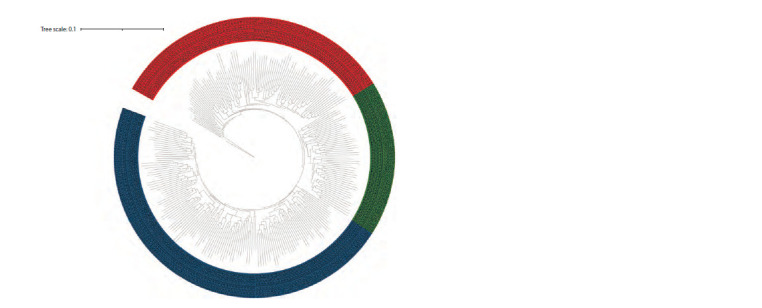
Phylogenetic tree of 272 barley samples constructed by the hierarchical clustering method by their genetic similarity estimated using the GBS data.

The phylogenetic tree constructed by the hierarchical clustering method is shown in Figure 5. Samples in the tree diagram are colored by cluster membership (see Fig. 4). It allows us to identify three large clusters in the population, which is consistent with the data presented in Figure 4.

## Discussion

The decreasing cost and time required for GBS sequencing has led to a large number of experiments performed by this method. For example, the IPK Gatersleben barley genetic profile database (Milner et al., 2019) contains 22,626 samples obtained by the GBS method. Such a large number of samples requires a fast and high-quality data processing method. To date, pipelines processing GBS results already exist. However, despite the qualitatively selected software packages and the possibility to adjust parameters to the organisms under study, these pipelines have some disadvantages. For example, GBS-SNP-CROP and TASSEL have no possibility to automate the calculation process (each step should be started manually), which significantly reduces the speed of the study. GB-eaSy does not allow simultaneous research of seve- ral libraries of raw reads at once. In all existing pipelines, there is no possibility to switch off an unnecessary or passed step. For example, if there is no way to provide barcode data for the libraries being examined, then none of the listed pipelines will work. Also, in most pipelines, there is no possibility of automatic installation of all necessary software packages.

**Fig. 6.**
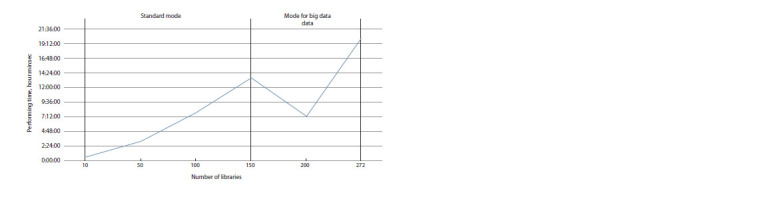
Dependence of time spent on the conveyor operation on the number of libraries under study.

The pipeline we developed is based on the method proposed in (Jayakodi et al., 2020). In this paper, the bioinformatics tools are selected in such a way as to provide the most accurate polymorphism search result. However, this method is well applicable for small data, up to 50 libraries; as the number of libraries increases, the load on RAM and the space occupied on the hard disk increases. This leads to errors and interruption of the computation process. Thus, we proposed an approach for large GBS data processing based on (Jayakodi et al., 2020) method. The results of this approach are summarised in Supplementary Material and Figure 6.

As can be seen from Figure 6, our proposed approach significantly speeds up the calculation process for large data, but for small data, the difference in calculation speed is not large. Therefore, this mode is activated only for the VCF file data with the total size exceeding 500 GB.

The proposed pipeline was built using the Snakemake workflow manager. This method of implementation allows to automatically take into account the completed tasks for each sample, which eliminates the duplication of tasks, and also allows to resume the calculation process from the moment of its last interruption (for example, due to an error). Modular structure allows for more convenient functionality of manipulation of the pipeline steps (exclusion, addition, switching off some steps). Snakemake also has the ability to automatically install all the necessary software for the pipeline.

## Conclusion

Genotyping by sequencing methods have demonstrated their reliability and flexibility for a number of plant species and populations. They have reduced both the cost and the time required to sequence the samples under study, which has allowed even more sequencing to be performed. In this work, we proposed a GBS-DP bioinformatics pipeline, which allows us to process large-scale sequencing data performed by the GBS method. The results demonstrate a fairly high speed of this pipeline for both large data (more than 400 libraries) and small data (~30 libraries). The pipeline also provides analysis of detected polymorphisms.

## Conflict of interest

The authors declare no conflict of interest.
